# Targeted Inhibition of Matrix Metalloproteinase-8 Prevents Aortic Dissection in a Murine Model

**DOI:** 10.3390/cells11203218

**Published:** 2022-10-14

**Authors:** Chengxin Zhang, Kaiyuan Niu, Meixia Ren, Xinmiao Zhou, Zhisheng Yang, Mei Yang, Xinxin Wang, Jun Luo, Yue Shao, Cheng Zhang, Dan Chen, Shan Gao, Shenglin Ge, Qingchen Wu, Qingzhong Xiao

**Affiliations:** 1 Department of Cardiovascular Surgery, First Affiliated Hospital of Anhui Medical University, No. 218, Jixi Road, Shushan District, Hefei 230022, China; 2 William Harvey Research Institute, Faculty of Medicine and Dentistry, Queen Mary University of London, London EC1M 6BQ, UK; 3 Department of Otolaryngology, The Third Affiliated Hospital of Anhui Medical University, Huaihe Road, Luyang District, Hefei 230061, China; 4 Shengli Clinical Medical College of Fujian Medical University, Fujian Medical University, Fuzhou 350005, China; 5 Department of Geriatric Medicine, Fujian Provincial Hospital, Fujian Key Laboratory of Geriatrics, Fujian Provincial Center for Geriatrics, Fuzhou 350001, China; 6 Department of Cardiothoracic Surgery, The First Affiliated Hospital of Chongqing Medical University, Chongqing 400016, China; 7 Department of Pharmacology, Basic Medical College, Anhui Medical University, Hefei 230032, China

**Keywords:** aortic dissection, matrix metalloproteinase-8, smooth muscle cell apoptosis, Angiotensin I, Angiotensin II, reactive oxygen species, inflammation

## Abstract

Aortic dissection (AD) is a lethal aortic pathology without effective medical treatments since the underlying pathological mechanisms responsible for AD remain elusive. Matrix metalloproteinase-8 (MMP8) has been previously identified as a key player in atherosclerosis and arterial remodeling. However, the functional role of MMP8 in AD remains largely unknown. Here, we report that an increased level of MMP8 was observed in 3-aminopropionitrile fumarate (BAPN)-induced murine AD. AD incidence and aortic elastin fragmentation were markedly reduced in MMP8-knockout mice. Importantly, pharmacologic inhibition of MMP8 significantly reduced the AD incidence and aortic elastin fragmentation. We observed less inflammatory cell accumulation, a lower level of aortic inflammation, and decreased smooth muscle cell (SMC) apoptosis in MMP8-knockout mice. In line with our previous observation that MMP8 cleaves Ang I to generate Ang II, BAPN-treated MMP8-knockout mice had increased levels of Ang I, but decreased levels of Ang II and lower blood pressure. Additionally, we observed a decreased expression level of vascular cell adhesion molecule-1 (VCAM1) and a reduced level of reactive oxygen species (ROS) in MMP8-knockout aortas. Mechanistically, our data show that the Ang II/VCAM1 signal axis is responsible for MMP8-mediated inflammatory cell invasion and transendothelial migration, while MMP8-mediated SMC inflammation and apoptosis are attributed to Ang II/ROS signaling. Finally, we observed higher levels of aortic and serum MMP8 in patients with AD. We therefore provide new insights into the molecular mechanisms underlying AD and identify MMP8 as a potential therapeutic target for this life-threatening aortic disease.

## 1. Introduction

Aortic dissection (AD) is a lethal aortic pathology with acute onset and minimal prior symptoms. AD is the striping of the intimal layer of the aorta, most commonly within the ascending or proximal descending aorta, with tearing of the elastic lamina, resulting in the creation of a false lumen within the effected portion of the vessel [1]. The incidence of AD stands at between 2.9 and 3.5 per 100,000 in the general population, increasing to 16 and 30~34 per 100,000 for those who are older than 65 and 75 years, respectively [1,2]. Classification of AD is mainly based on anatomical location, with Stanford type A dissections involving the ascending aorta whereas type B dissections do not involve the ascending aorta [1,2,3]. Type A acute AD (14 days post-onset) is a challenging clinical emergency with an exceptionally high mortality (26% and 58% for patients who were managed surgically or receiving non-surgical cares, respectively) [3]. Despite type B acute AD generally being more benign and medical management for the associated symptoms able to improve a patient’s clinical outcome, a substantial proportion of patients still encounter multiple catastrophic events within 1 week post-onset [4]. Although hypertension, old age, atherosclerosis, previous cardiovascular surgery, inherited disorders, and aortic vasculitis have been suggested as the associated risk factors for AD [5,6], the underlying pathological mechanisms responsible for triggering the disease remain elusive. A hallmark histological finding associated with AD is cystic medial necrosis, characterized by decreased vascular smooth muscle cells (SMCs), increased cell apoptosis, mucoid deposition, elastin deficiency, and fragmentation [5]. However, the direct cellular and molecular mechanism that link the medial degeneration and the onset of AD has not been fully elucidated. 

Matrix metalloproteinase-8 (MMP8) regulates important cellular functions and signal pathways, mainly through its potent proteolytic activity on matrix proteins, including fibrillar collagens, laminin, fibronectin [7], and fibromodulin [8]. Importantly, MMP8 also cleaves many other important regulatory proteins, such as chemokines (e.g., CXCL5 [9] and CXCL11 [10]), Angiotensin I (Ang I) [11], A disintegrin and metalloproteinase domain-containing protein 10 (ADAM10) [12,13], and transforming growth factor-β (TGF-β) [8], thereby activating or inactivating these regulatory proteins. Clinical studies have suggested a role for MMP8 in cardiovascular diseases [14,15,16]. Interestingly, apart from neutrophils, MMP8 was reported to be expressed in multiple other cells in atherosclerotic lesions, including macrophages, SMCs, endothelial cells (EC) [17], and stem/progenitor cells [13]. By generating MMP8-deficient mice, we were the first to confirm the critical role of MMP8 in the pathogenesis of atherosclerosis [11], bone marrow-derived stem/progenitor cell migration and recruitment into atherosclerotic lesions [13], angiogenesis [18], vascular injury-induced neointima formation [12], macrophage differentiation and polarization [8], and adventitia stem/progenitor cell differentiation [19]. However, the potential role of MMP8 in AD remains to be determined. In the current study, MMP8 genetic-deficient mice (Apolipoprotein E^−/−^/MMP8^−/−^ mice), as generated in our previous study [11], and a specific MMP8 inhibitor (MMP8i, CAS 236403-25-1) were employed in this study to establish the causal role of MMP8 in thoracic AD (TAD) and explore the therapeutic potential of MMP8i in treating TAD. A variety of biochemical assays were used to elucidate the molecular mechanisms underlying MMP8’s involvement in TAD.

## 2. Materials and Methods

### 2.1. Materials

Antibodies against alpha smooth muscle actin (SMA, rabbit IgG, ab5694), MMP8 (rabbit IgG, ab53017), CD68 (rabbit IgG, ab125212), VCAM1 (rabbit IgG, ab134047), 8-Hydroxy-2**′**-deoxyguanosine (8-OHdG, mouse IgG, ab48508), and VCAM1 (rabbit IgG, ab134047) were purchased from Abcam, Cambridge, UK. Antibody against Angiotensin I (mouse IgG, sc-74528) was from Insight Biotechnology, UK. Antibody against Angiotensin II (rabbit IgG, ABIN6993662) was from Antibodies-online, Germany. Antibody against Ly6G (Rat IgG, 127602) was from Biolegend, London, UK. Monoclonal anti-α smooth muscle actin (SMA) (Clone 1A4, mouse IgG, A5228) was from Merck, Dorset, UK. All secondary antibodies and other materials were from ThermoFisher Scientific, Cheshire, UK, unless specifically indicated.

### 2.2. Animal Experiments, Anesthesia, and Euthanasia

All animal experiments were conducted according to the Animals (Scientific Procedures) Act of 1986 (United Kingdom). All the animal procedures were approved by Queen Mary University of London’s ethics review board (PPL number: PP5521236), and conform to the guidelines from Directive 2010/63/EU of the European Parliament on the protection of animals used for scientific purposes and the NIH guidelines (guide for the care and use of laboratory animals). For the 3-aminopropionitrile fumarate (BAPN)-induced TAD model, three-week-old mice (WT: ApoE^−/−^/MMP8^+/+^ or MMP8_KO: ApoE^−/−^/MMP8^−/−^) of both sexes were fed a normal diet and randomly administered with vehicle (water) or freshly prepared BAPN (A3134, Merck, Dorset, UK) solution dissolved in the drinking water (0.25% *wt/vol*) for up to 4 weeks, as described previously [20,21,22,23]. To determine the therapeutic potential of MMP8, mice were randomized to receive either intraperitoneal injections of vehicle (1% DMSO) or the specific MMP8i, (3R)-(+)-[2-(4-methoxybenzenesulfonyl)-1,2,3,4-tetrahydroisoquinoline-3-hydroxamate (CAS Number: 236403-25-1(444237, Merck, Dorset, UK, dissolved in 1% DMSO), as reported in previous studies [24,25,26]. Daily injections of vehicle or MMP8i at a concentration of 5 mg/kg per day for up to 28 days, starting 1 day prior to BAPN administration. At the end of the protocol, all mice were euthanized by placing them under deep anesthesia with 100% O_2_/5% isoflurane, followed by decapitation.

### 2.3. Collection of Human Aorta Tissue Specimens and Sera

Human ascending aortic tissue specimens and sera were obtained from consented patients at the time of elective surgery through a protocol approved by the Institutional Review Board (IRB) at the First Affiliated Hospital of Chongqing Medical University between October 2020 and May 2021 (research ethics approval reference number: 2018-022-2), as previously described [27]. Briefly, 22 human ascending aortic tissues with dissection were collected from type A AD patients who were free from connective tissue disorders, such as Turner’s, Loeys–Dietz, Ehlers–Danlos, and Marfan’s syndrome during surgical operations, and from 12 healthy ascending aortic tissues without dissection, collected from organ donors who were free of any known aortic diseases. Meanwhile, serum was collected from age- and sex-matched acute TAD patients and normal heathy subjects and kept at −80 °C for future use. All patients gave their written, informed consent prior to inclusion in the study, and all experiments were conducted according to the principles expressed in the Declaration of Helsinki. The basic clinical characteristics of the study population are described in Supplementary Tables S1 and S2.

### 2.4. Histopathological Analysis

Histopathological analysis was described in our previous studies [13,28]. Briefly, the ascending thoracic aorta was fixed with 10 % formalin for 24 h at room temperature before being embedded in paraffin for sectioning. Tissues were sectioned at 5 μm and underwent HE staining and elastin Van Gieson (EVG) staining, respectively. Degradation of medial elastic lamina was analyzed by EVG staining using Elastic Stain Kit (Verhoeff Van Gieson) (Abcam, Cambridge, UK, ab150667) as per manufacturer instructions. Images were taken and elastin breaks (fragmentation) per section were manually counted.

### 2.5. Blood Pressure Measurement

Blood pressure was measured in conscious mice using a volume-pressure-recording sensor and occlusion tail cuff (Coda2, Kent Scientific, Torrington, CT 06790, USA), as described in our previous study [11].

### 2.6. Aortic Tissue Immunofluorescence Staining

The procedure used for aortic immunofluorescence staining was similar to that described in our previous studies [12,29,30,31]. Briefly, paraffin thoracic aortic sections were deparaffined with xylene and rehydrated with ethanol, followed by antigen retrieval in a Universal HIER antigen retrieval reagent (Abcam, Cambridge, UK, ab208572). After blocking the sections, they were incubated with the indicated primary antibodies or respective IgG controls diluted in blocking buffer in a cold room (4 °C) overnight. The tissue sections were then washed and subsequently incubated with an appropriate Alexa Fluor Plus 2nd antibody (1:1000 dilution), followed by nuclei staining with 4,6-diamidino-2-phenylindole (DAPI) (1 ug/mL). After mounting, the slides were examined using a laser scanning confocal microscope (Zeiss LSM 510 Mark 4) and Zen 2009 image software. The mean fluorescence intensity (MFI) for target proteins and DAPI of the aortic wall from each section were measured with Image J pro software by two experienced investigators blinded to the treatments, and presented as the relative MFI (target proteins over DAPI). Alternatively, cells stained positive for the target proteins and DAPI (total cells) were counted by two experienced investigators blinded to the treatments. Three sections were analyzed per vessel sample and averaged.

### 2.7. TUNEL Staining for SMC Apoptosis

As described previously [32,33], cell apoptosis was detected in cultured SMCs with the indicated treatments, as shown in the respective figure legend, using a commercial Terminal deoxynucleotidyl transferase dUTP nick end labeling (TUNEL) Assay Kit (11684795910, Sigma, Dorset, UK), according to the manufacturer’s instructions. Cells were co-stained with DAPI. TUNEL+ cells and DAPI-stained cells were counted as apoptotic cells and total cells, respectively. For aortic SMC apoptosis, paraffin aortic sections were deparaffined with xylene and rehydrated with ethanol, followed by TUNEL staining analysis according to the manufacturer’s instructions. Co-staining of TUNEL with SMA was performed to detect apoptotic SMC in the aorta. TUNEL-positive cells over total cells were counted by two experienced investigators blinded to the treatments. 

### 2.8. Bone Marrow Cell Isolation 

Monocyte [13,28] and neutrophils [34] were isolated from WT and MMP8_KO bone marrow, as described previously. Briefly, bone marrow cells were harvested from mouse femurs and tibias and re-suspended in Red Blood Cell Lysis Buffer (Sigma, Dorset, UK, 11814389001) to lyse the red blood cells. Mouse bone marrow monocytes were isolated from the unlysed cells using a Monocyte Isolation Kit (Miltenyi Biotec, Woking, UK, 130-100-629), as per the manufacturer’s instructions, while mouse bone marrow neutrophils were isolated using Histopaque-based density gradient centrifugation (Sigma, Dorset, UK, 1077/1119). The isolated cells were re-suspended in RPMI 1640 supplemented with 1% penicillin/streptomycin and subjected to the respective analysis as indicated in the figure legend. 

### 2.9. C166 Culture and MISSION esiRNA Transfection

C166 cells, a widely used mouse endothelial cell line, were purchased from ATCC (CRL-2581) and maintained in Dulbecco’s Modified Eagle’s Medium (DMEM) supplemented with 10 % FBS and 1 % penicillin/streptomycin, as per the manufacturer’s instructions. Control scramble esiRNA or Vcam1-specific esiRNA (50 nM, final concentration) were transfected into C166 cells using TransIT-X2 Transfection Reagent (Geneflow Limited, Lichfield, UK), according to the manufacturer’s instructions, and as previously described [33]. MISSION esiRNA is a heterogeneous mixture of siRNAs that all target the same mRNA sequence, resulting in highly specific and effective gene silencing. All esiRNAs (EHUEGFP for si-NT, and EMU009991 for si-Vcam1) were purchased from Sigma. With an optimum condition (clean and healthy C166 cells at exponential growth phase with 50~70% confluent), a satisfactory transfection efficiency (>80%) is normally achieved in our laboratory using the transfection protocols.

### 2.10. Murine Aortic SMC Culture and Treatments

Primary murine aortic SMCs were isolated from eight-week-old WT or MMP8_KO mice with both sexes, and routinely maintained in DMEM supplemented with 10% FBS, as described in our previous studies [32,33,35,36,37]. Aortic SMCs between Passages 3 (P3) and 8 (P8) were used in our study, as described previously [38]. Aortic SMCs were serum-starved for 24~48 h (0% FBS), followed by various treatments, as indicated, for up to 48 h.

### 2.11. Real-Time Quantitative PCR (RT-qPCR) 

RT-qPCR was performed as previously described [32,33,37,38]. Briefly, total RNA was extracted from murine aortas or cells using Trizol reagent (Sigma, Dorset, UK) according to the manufacturer’s instructions and subjected to DNase I (Sigma, Dorset, UK) digestion to remove potential DNA contamination. cDNA was reversely transcribed from total RNAs using an Improm-II^TM^ RT kit (Promega, Madison, WI, USA) with RNase inhibitor (Promega, Southampton, UK), and Random primers (Promega, Southampton, UK), and diluted to a working concentration of 5 ng/μL. FS UNIVERSAL SYBR GREEN MASTERROX was used in RT-qPCR. The relative mRNA expression level was defined as the ratio of target gene expression level to 18S expression level, with that of the control sample set as 1.0. Primers were designed using Primer Express software (Applied Biosystems) and the sequence for each primer was listed in Supplementary Table S3.

### 2.12. ELISA Analysis

Plasma MMP8, Ang I, and Ang II were measured using a Mouse MMP8 ELISA Kit, (ab206982, Abcam, Cambridge, UK) and Angiotensin I/II ELISA kit (Enzo Life Sciences, Exeter, UK, ADI-900-203/ADI-900-204), according to the manufacturer’s instructions.

### 2.13. MMP8 Activity Analysis

MMP8 activity was measured using a SensoLyte**^®^** 520 MMP-8 Assay Kit (ABIN1882526, antibodies-online GmbH, Aachen, Germany), as per the manufacturer’s instructions. Briefly, 50 µL of aortic lysates or cell culture supernatant were incubated with 5-FAM (fluorophore)/QXL520™ (quencher)-labelled substrates, fluorescence resonance energy transfer (FRET) peptide substrates, for 1 h in a black 96-well plate at room temperature in a dark room. The recovered fluorescence of 5-FAM by MMP8, representing MMP8 activity, was continuously monitored and measured at excitation/emission of 490 nm/520 nm, using a Tecan microplate reader (Tecan Trading AG, Männedorf, Switzerland). The relative fluorescence unit (RFU) was calculated by subtracting blank fluorescence readings from all measurements (control and treatments).

### 2.14. ROS Measurement

Reactive oxygen species (ROS) levels in cultured aortic SMCs were measured using Cellular Reactive Oxygen Species Detection Assay Kit (ab186027, Abcam, Cambridge, UK), according to the manufacturer’s instructions. Briefly, cultured SMCs were serum-staved for 24 h, followed by incubation with ROS Red Working Solution for one hour in a 37 °C/5% CO_2_ incubator. After then, cells were incubated with BAPN (25 μg/mL), Ang I (10 nM), and/or Ang II (10 nM), as indicated in the figures, for another hour in a 37 °C/5% CO_2_ incubator to induce ROS generation. Fluorescence was measured at excitation/emission of 520 nm/605 nm using a Tecan microplate reader (Tecan Trading AG, Männedorf, Switzerland). Relative fluorescence unit (RFU) was calculated by subtracting blank fluorescence readings from all measurements (control and treatments).

### 2.15. Cell Invasion and Transendothelial Migration Assay 

Similar to our previous study [13], bone marrow monocytes or neutrophils isolated from WT and MMP8_KO mice were subjected to cell invasion assays using the QCM ECMatrix Cell Invasion Assay kit (Merck/Sigma, Dorset, UK ECM550), according to the manufacturer’s instructions. Briefly, 1 × 10^5^ neutrophils/monocytes in 200 μL serum-free RMPI 1640 medium supplemented with 25 μg/mL BAPN were placed over the inner chamber of inserts in a 24-well tissue culture plate and 500 μL serum-free RMPI 1640 medium supplemented with 1% BSA and macrophage inflammatory protein 2 (MIP2, 100 ng/mL, for neutrophils) or monocyte chemoattractant protein-1 (MCP-1, 100 ng/mL for monocytes) were added into the bottom chamber of the insert. Plates were incubated at 37 °C for 4 h. The invasive cells on the lower surface of the membrane were stained with 500 μL of the staining solution for 20 min. After then, the stained cells were dissolved in 100 μL 10% acetic acid, and absorbance (OD_560_) was detected at 560 nm using a Tecan microplate reader (Tecan Trading AG, Männedorf, Switzerland). 

For the transendothelial migration assay, polycarbonate membrane inserts (8-μm pore size; Greiner Bio-One Inc., Stonehouse, UK) were used. One hundred thousand neutrophils/monocytes in 200 μL serum-free RMPI 1640 medium supplemented with 25 μg/mL BAPN, in the absence or presence of 10 nM Ang I or Ang II, were placed over the inner chamber of inserts pre-grown with a monolayer of C166 cells in a 24-well tissue culture plate, and 500 μL serum-free RMPI 1640 medium supplemented with 1% BSA and 100 ng/mL MIP2 or MCP-1, as indicated in the respective figures, was added into the bottom chamber of the insert. Plates were incubated at 37 °C for 4 h. The cells that had migrated through to the lower surface of the insert were scraped off and mixed with the cells that had migrated into the bottom well. After being collected, the migrated cells were stained, lysed, and measured as mentioned above. 

### 2.16. Cell Viability Analyses 

WT and MMP8_KO aortic SMCs (0.75 × 10^4^ per well) cultured in 96-well plates overnight were subjected to serum starvation for 48 h, followed by various treatments, as indicated in figure legend, for up to 48 h. Cell viability was evaluated using the Cell Counting Kit-8 (CCK-8) kit (Sigma/Merck, Dorset, UK 96992-500TESTS-F), according to the manufacturer’s instructions, and as described previously [35,36]. The absorbance of the samples representing cell viability was measured by a microplate reader at 450 nm (OD_450_). 

### 2.17. Statistical Analysis

Age- and sex-matched (male:female = 2:1) animals were randomly allocated to their experimental groups. Data collection and evaluation of all experiments were performed blinded to the group identity. Since we observed no apparent sexual dimorphism in BAPN-induced TAD formation in ApoE^−/−^ mice (data not shown), data from both sexes were pooled for statistical analysis in this study. Results are presented as the mean ± standard error of the mean (SEM). Statistical analysis was performed using GraphPad Prism (v9.1, GraphPad Software, San Diego, California, USA). The Shapiro–Wilk normality test and an F-test were used for checking the normality and homogeneity of variance of the datasets, respectively. Accordingly, two tailed an unpaired Student’s t-test was used for comparisons between two groups, or one/two-way analysis of variance with a post-hoc Tukey test was applied when more than two groups were compared if the data displayed a normal distribution and homogeneity of variance. Conversely, non-parametric Mann–Whitney U tests were applied for comparing two groups if the data did not display a normal distribution. Additionally, a Log-rank (mantel-cox) test and Chi-square test were applied to compare the survival rates and TAD incidence among different groups, respectively. Alpha = 0.05 was chosen as the significance level, and a value of *p* < 0.05 was considered as statistically significant.

## 3. Results

### 3.1. MMP8 Expression Was Increased during BAPN-Induced TAD Development

To study the direct cellular and molecular mechanism underlying TAD, a well-established BAPN-induced murine TAD model [20,21,22,23] was adapted in this study. Similar to previous studies [20,21,22,23], BAPN administration through drinking water (0.25%, *wt/vol*) caused approximately 14% and 40% mortality at 3 and 4 weeks post-treatment, respectively (Figure S1A–C). Histopathological analysis with elastin Van Gieson (EVG) staining showed that while no TAD occurred in mice that received either vehicle or BAPN treatment for 1 and 2 weeks, TAD was observed in two out seven (2/7) and seven out of ten (7/10) mice that received BAPN treatment at 3 and 4 weeks post BAPN administration, respectively (Figure S1B,D). We found that TAD mainly occurred in the ascending aorta or at the proximal site of the descending thoracic aorta (Figure S1A). We also observed increasing numbers of elastin breaks (fragmentation) in a time-course-dependent manner (Figure S1B,E). Importantly, compared with mice that received the vehicle, both MMP8 aortic gene expression (Figure S1F) and plasma levels (Figure S1G) were significantly upregulated during BAPN-induced TAD formation, which peaked at 1 week post-BAPN administration and was maintained at a higher expression level over the 4-week period. Interestingly, such upregulation appears to precede TAD onset, which was normally observed during the third week of BAPN administration (Figure S1B,D), inferring an involvement of MMP8 in TAD onset and formation. 

### 3.2. MMP8 Deficiency Inhibited BAPN-Induced TAD 

**Figure 1:**
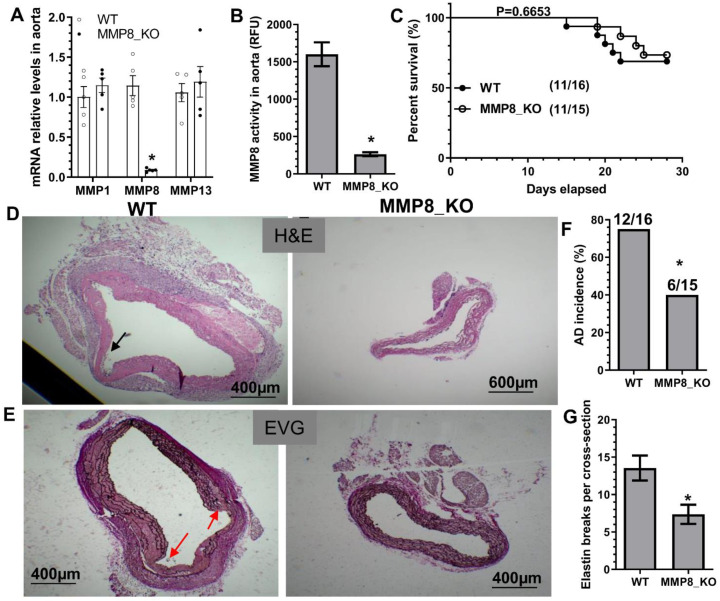
A causal role for MMP8 in BAPN-induced TAD formation. Three-week-old MMP8-knockout mice (MMP8_KO, ApoE^−/−^/MMP8^−/−^) and their wildtype control littermates (WT, ApoE^−/−^/MMP8^+/+^) administered with BAPN in drinking water for two (**A**,**B**) or four (**C**–**G**) weeks, respectively. (**A**) Thoracic aortic gene expression was analyzed by RT-qPCR. (**B**) Thoracic aortic MMP8 activity was measured using SensoLyte**^®^** 520 MMP-8 Assay Kit. (**C**) Animal survival rate (Log-rank (Mantel–Cox) test). Images for HE staining (**D**), EVG staining (**E**), and the quantitative data of AD incidence (**F**) and elastin breaks (**G**) are presented here. Note: Thoracic AD incidence was defined by the mice that died from thoracic aortic rupture, and mice identified with one or more aortic pathologies (aortic intima tear, false lumen, and intramural hematoma). Black or red arrows indicate AD or intima tearing. Data presented here are representative (**D**,**E**) or the mean ± SEM of five (**A**,**B**) or eleven mice (**C**–**G**), respectively (*n* = 5 or 11 mice). * *p* < 0.05 (versus WT, unpaired *t*-test in **A**,**B**,**G**; Chi-square test in **F**).

To study the causal role of MMP8 in TAD, both MMP8-knockout mice (MMP8_KO, ApoE^−/−^/MMP8^−/−^) and their wildtype control littermates (WT, ApoE^−/−^/MMP8^+/+^) were subjected to BAPN administration. As expected, the MMP8 aortic expression level was absent or extremely low in MMP8_KO mice, while the aortic expression levels of the other two collagenases, MMP1 and MMP13, were unchanged in MMP8_KO mice (Figure 1A). Similarly, MMP8_KO mice exhibited an extremely low level of aortic MMP8 activity upon BAPN administration (Figure 1B). No significant difference in terms of mortality rate or aortic rupture was observed between WT and MMP8_KO mice (Figure 1C), while a lower TAD incidence and decreased levels of elastin breaks (Figure 1D–G) were observed in MMP8_KO mice upon BAPN treatment, confirming a causal role for MMP8 in TAD formation.

### 3.3. Pharmacological Inhibition of MMP8 Decreased TAD 

**Figure 2:**
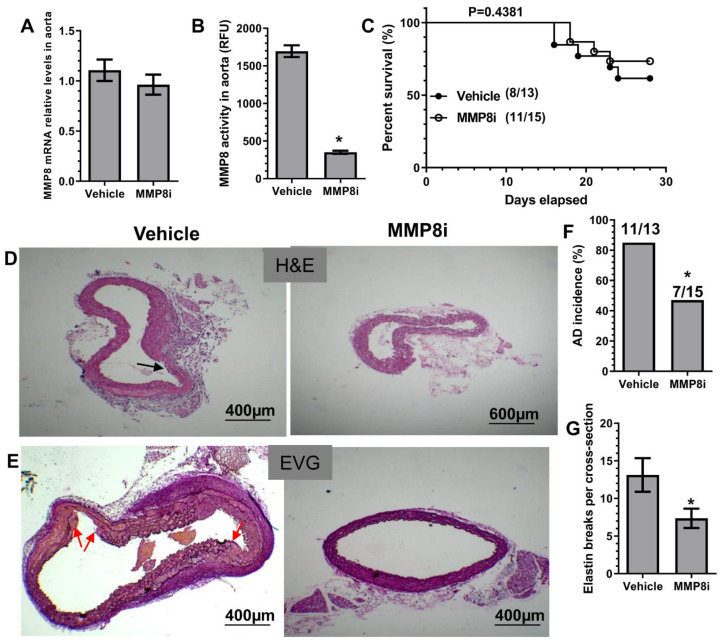
Pharmacological inhibition of MMP8 decreased BAPN-induced TAD formation. Three-week-old WT mice administered with BAPN were randomly injected with vehicle (1% DMSO) or the specific MMP8 inhibitor (MMP8i, CAS Number: 236403-25-1, 5 mg/kg per day) for two (**A**,**B**) or four (**C**–**G**) weeks, respectively. Thoracic aortic MMP8 gene expression (**A**) and activity (**B**) were analyzed by RT-qPCR and SensoLyte**^®^** 520 MMP-8 Assay Kit, respectively. (**C**) Animal survival rate (Log-rank (Mantel–Cox) test). Images for HE staining (**D**), EVG staining (**E**), and the quantitative data of AD incidence (**F**) and elastin breaks (**G**) are included here. Black or red arrows indicate AD or intima tearing. Data presented here are representative (**D**,**E**) or the mean ± SEM of five (**A**,**B**) or eight (vehicle)/eleven (MMP8i) mice (**C**–**G**), respectively (*n* = 5 or 8/11 mice). * *p* < 0.05 (versus vehicle, unpaired *t*-test in **B**,**G**; Chi-square test in **F**).

To explore a therapeutic value of MMP8 inhibition in BAPN-induced TAD formation, we treated WT mice with an in vivo potent and highly specific MMP8 inhibitor (MMP8i, CAS 236403-25-1), as previously described [24,25,26]. While aortic MMP8 gene expression was not affected by MMP8i treatment (Figure 2A), it caused a significant decrease in aortic MMP8 activity (Figure 2B). Similar to MMP8 gene deletion, MMP8i treatment caused no apparent change in survival rate (Figure 2C) but resulted in decreased levels of TAD incidence and elastin fragmentation (Figure 2D–G), demonstrating its therapeutic value in TAD treatment through pharmacological inhibition of MMP8.

### 3.4. Aortic Inflammatory Cell Accumulation and SMC Apoptosis Was Reduced in MMP8_KO Mice

**Figure 3:**
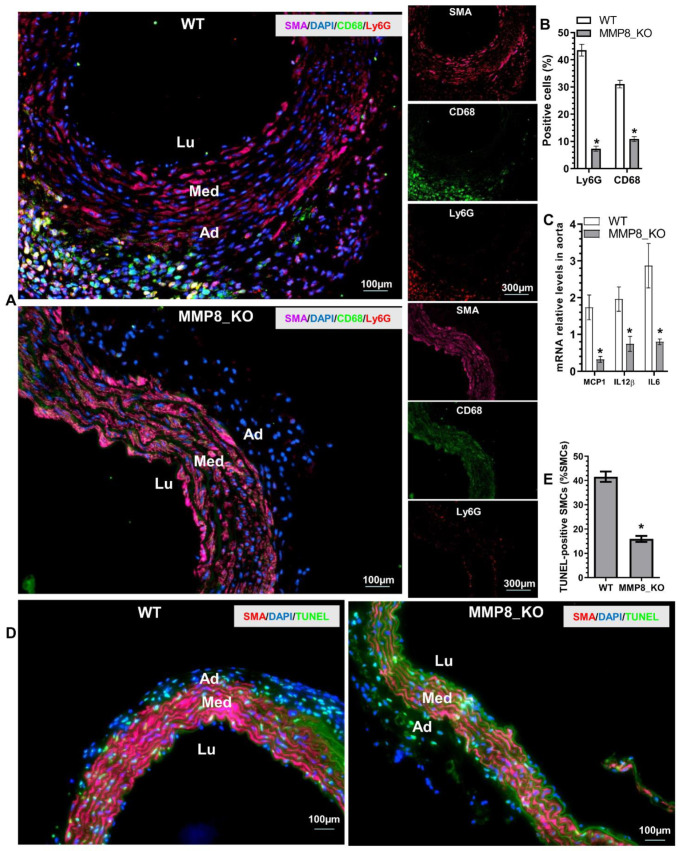
Reduced aortic inflammatory cell accumulation and SMC apoptosis were observed in MMP8_KO mice. Three-week-old WT and MMP8_KO mice were administered with BAPN in drinking water for two weeks, after which thoracic aortic tissues were collected and subjected to histological analysis. (**A**,**B**) Immunofluorescent staining analysis of the inflammatory cells in the aorta. (**C**) RT-qPCR analysis of inflammatory gene expression in the thoracic aorta. (**D**,**E**) TUNEL staining analysis of SMC apoptosis in the thoracic aorta. Data presented here are representative (**A**,**D**) or the mean ± SEM of six (**B**,**C**,**E**) mice, respectively (*n* = 6 mice). * *p* < 0.05 (versus WT, unpaired *t*-test). Lu, lumen; Med, media, Ad, adventitia.

Immunofluorescent (IF) staining was conducted in thoracic aortic tissues from mice that received BAPN treatment for two weeks, when TAD onset rarely occurred, as shown in Figure S1C. IF staining data showed that a large number of Ly6G^+^ neutrophils and CD68^+^ macrophages were accumulated in the adventitia of WT aortas, while much lower numbers of these inflammatory cells were observed in MMP8_KO adventitia (Figure 3A,B). Similarly, inflammatory gene expressions were significantly decreased in MMP8_KO aortas (Figure 3C). Moreover, TUNEL staining revealed that SMC apoptosis was much lower in MMP8_KO aortas than that in WT aortas (Figure 3D,E). Interestingly, IF staining analysis with antibodies against SMA, Ly6G, and MMP8 in the aortic tissues obtained from WT mice administrated with BAPN for three weeks showed that a large number of Ly6G^+^ neutrophils expressing MMP8 were observed within the media layer and at the dissection sites (Figure S2). These data strongly suggest a role for MMP8 in inflammatory cell migration and recruitment into aortic walls upon BAPN treatment. 

### 3.5. Reduced Ang II Levels, Lower Blood Pressure, and Decreased VCAM-1 Expressions Were Observed in MMP8_KO Mice

**Figure 4:**
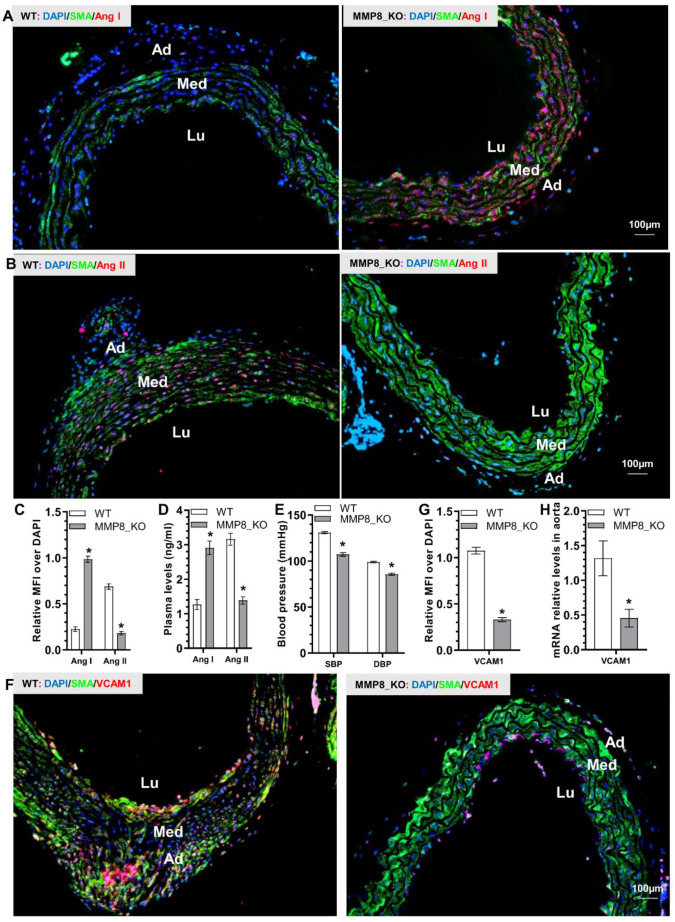
Reduced Ang II levels, lower blood pressure, and decreased VCAM-1 expressions were observed in MMP8_KO mice. Three week-old WT and MMP8_KO mice were administered with BAPN in drinking water for two weeks, thoracic aortic tissues and plasma were collected for analysis. (**A**–**C**) Immunofluorescent staining analysis of the Ang I and Ang II expressions in thoracic aorta. (**D**) ELISA analysis of plasma Ang I and Ang II levels. (**E**) Systolic (SBP) and diastolic (DBP) blood pressure measurements. (**F**,**G**) Immunofluorescent staining analysis of the VCAM1 expressions in thoracic aorta. (**H**) RT-qPCR analysis of aortic VCAM1 gene expression. Data presented here are representative (**A**,**B**,**F**) or Mean ± S.E.M of six (**C**–**E**,**G**–**H**) mice, respectively (*n* = 6 mice). * *p* < 0.05 (versus WT, unpaired *t*-test). Lu, lumen; Med, media, Ad, adventitia.

Since we have previously demonstrated that during hyperlipidemia-induced atherosclerosis MMP8 can cleave Angiotensin I (Ang I) to mainly generate Ang II, which up-regulates VCAM1 gene expression in endothelium and promotes leukocyte rolling and adhesion on vascular endothelium [11], we wondered if a similar mechanism underlying MMP8-mediated inflammatory cell accumulation, aortic inflammation and TAD formation. Indeed, we observed a much higher Ang I expression level, but a much lower Ang II expression level in MMP8_KO aorta than that in WT aorta (Figure 4A–C and Figure S3). A similar trend was observed with plasma Ang I and Ang II levels in MMP8_KO mice (Figure 4D). Expectedly, MMP8_KO mice exhibited lower levels of both systolic (SBP) and diastolic (DBP) blood pressure than that of WT mice (Figure 4E). IF staining analysis of the aortic tissues with antibodies against SMA and VCAM1 revealed a significant decrease of VCAM1 protein expression in MMP8_KO aorta (Figure 4F,G), which was further confirmed at mRNA expression level (Figure 4H). The above data suggest that MMP8 converts Ang I to Ang II, which increases blood pressure and up-regulates aortic VCAM1 upon BAPN treatment.

### 3.6. MMP8 Augmented Inflammatory Cell Invasion and Transendothelial Migration

**Figure 5:**
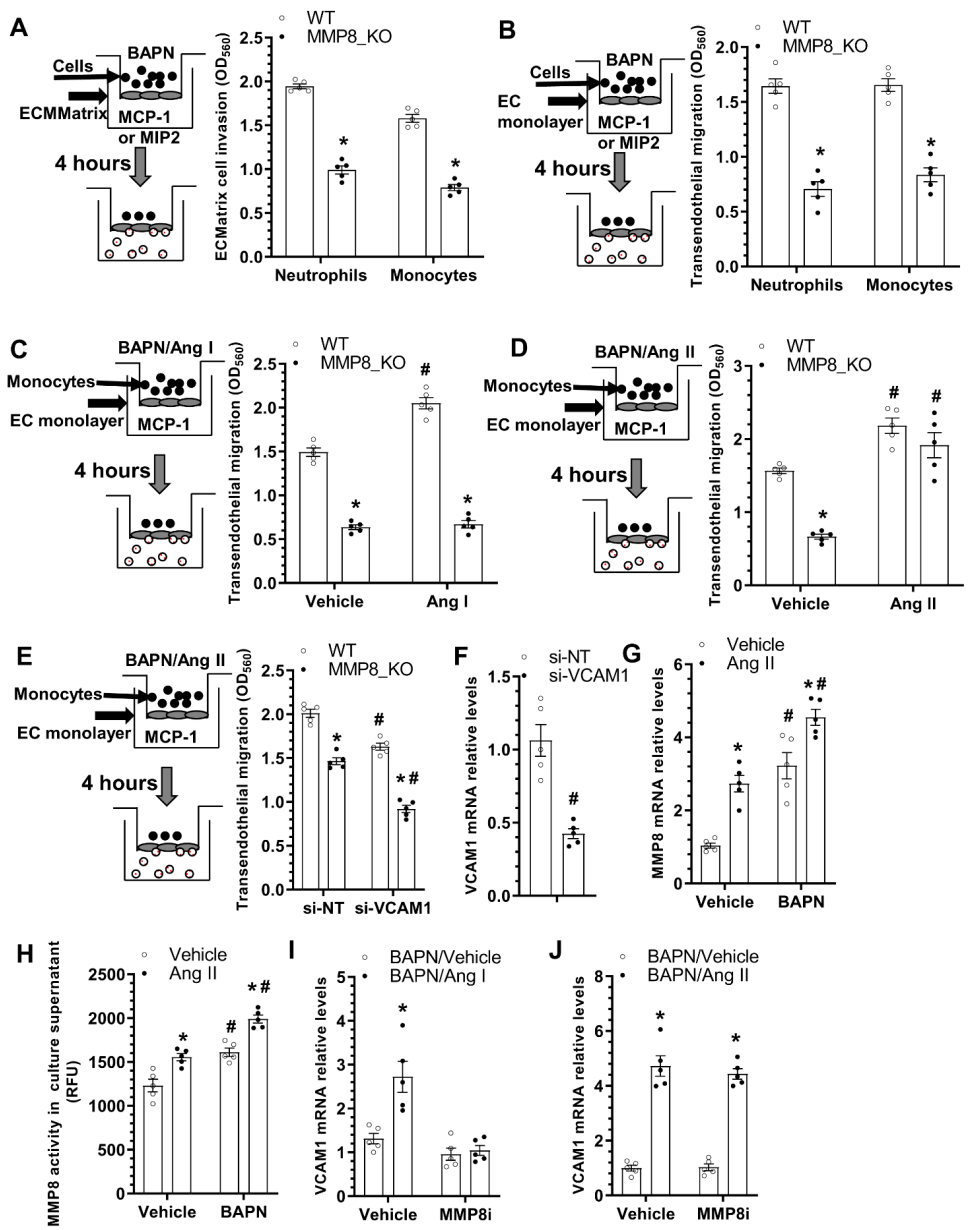
MMP8-deficiency causes decreased inflammatory cell invasion & transendothelial migration. Bone marrow neutrophils or monocytes were isolated from WT or MMP8_KO mice, and subjected to cell invasion (**A**) and transendothelial migration (**B**–**E**) assays, respectively. (**A**) The invasion capacity of cell was detected using QCM ECMatrix Cell Invasion Assay. (**B**) Cell transendothelial migration analysis. Mouse endothelial cells (EC, C166 cells) were pre-cultured onto transwell inserts (pore size: 8 µm) to form an EC monolayer, followed by transendothelial assays in response to 100 ng/mL macrophage inflammatory protein 2 (MIP2) for neutrophils or monocyte chemoattractant protein-1 (MCP-1) for monocytes, respectively. (**C**) Ang I increased WT but not MMP8_KO monocyte transendothelial migration. (**D**) Ang II promoted both WT and MMP8_KO monocyte transendothelial migration. (**E**,**F**) VCAM1 gene knockdown decreased monocyte transendothelial migration. C166 cells were transfected with control (si-NT) or VCAM1 specific (si-VCAM1) siRNAs, followed by transendothelial migration (**E**) and RT-qPCR (**F**) assays, respectively. (**G**) RT-qPCR analysis of MMP8 gene expressions in C166 cells with indicated treatment for 24 h. (**H**) MMP8 activity in cell culture supernatant with the indicated treatments for 24 h. (**I**,**J**) RT-qPCR analysis of VCAM1 gene expression in C166 cells with indicated treatments (25 μg/mL BAPN with or without 10 nM Ang I/Ang II) for 24 h. Data presented here are Mean ± S.E.M of five independent experiments (*n* = 5). * *p* < 0.05 (versus WT, or vehicle); ^#^ *p* < 0.05 (versus vehicle or si-NT); two-way ANOVA with a post-hoc Tukey test.

We have previously shown that MMP8 promotes stem/progenitor cell migration and recruitment into atherosclerotic lesions [13]. Moreover, abovementioned data (Figure 3 and Figure S2) also suggest MMP8 increases inflammatory cell accumulation within adventitia and promotes these cells migrating into media layer upon BAPN administration. We therefore further examined a functional role for MMP8 in inflammatory cell migration/recruitment. Data from QCM ECMatrix Cell Invasion Assay showed that MMP8 deficiency in both neutrophils and monocytes led to decreased extraceullar invasion capacity in response to macrophage inflammatory protein 2 (MIP2) or monocyte chemoattractant protein-1 (MCP-1) stimulations, respectively (Figure 5A). BAPN was included in all the migration experiments to mimic the pathological condition of the BAPN-induced TAD. EC monolayer was incorporate into the migration settings to further recapitulate the in vivo pathological environment of TAD. Similarly, a much lower transendothelial migration ability was observed in MMP8_KO neutrophils and monocytes (Figure 5B). These data have collectively demonstrated an important role for MMP8 in inflammatory cell invasion and migration under TAD pathological conditions. Interestingly, we observed that while Ang II could promote both WT and MMP8_KO monocytes transendothelial migration, Ang I only augmented the transendothelial migration capacity of WT monocytes (Figure 5C,D), suggesting that conversion of Ang I to Ang II by MMP8 is one of the molecular mechanisms underlying MMP8-mediated inflammatory cell migration during BAPN-induced TAD formation. Moreover, the transendothelial migration ability of WT monocytes was significantly impaired by VCAM1 knockdown in ECs, with further impairment was observed in MMP8_KO monocytes (Figure 5E,F). Further mechanistic studies showed that BAPN or Ang II could significantly up-regulate MMP8 expression in ECs, which was further increased by Ang II and BAPN combinational incubation (Figure 5G). A similar phenomenon was observed with MMP8 activity in EC culture supernatant (Figure 5H). Ang I dramatically increased VCAM1 gene expression in ECs, but such regulatory effect was absent when MMP8 was inhibited (Figure 5I). As expected, Ang II could activate VCAM1 gene expression in ECs regardless of MMP8 inhibition (Figure 5J). Taken together, the above data demonstrates that MMP8-mediated Ang II generation from Ang I is responsible for MMP8-mediated inflammatory cell migration/accumulation into aortic wall during BAPN-induced TAD formation.

### 3.7. MMP8 Mediated BAPN/Ang II-Induced SMC Inflammation and Apoptosis 

**Figure 6:**
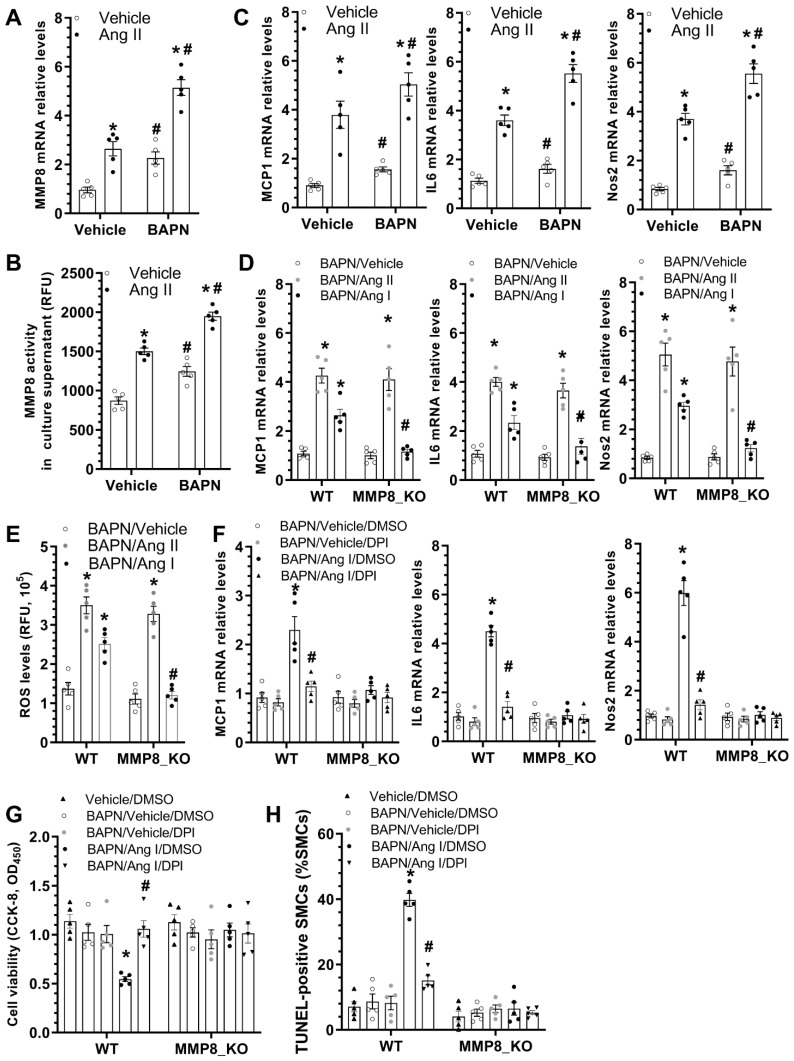
MMP8 increased BAPN/Ang II-induced SMC inflammation and apoptosis via ROS generation. (**A**,**B**) BAPN and Ang II synergically increased MMP8 gene expression and activity in SMCs. SMCs were treated with 25 μg/mL BAPN with or without 10 nM Ang II for 24 h. Total RNAs and cell culture supernatant were collected and subjected to RT-qPCR analysis (**A**) and MMP8 activity assay (**B**), respectively. (**C**) BAPN and Ang II synergically upregulated inflammatory gene expression. (**D**) Ang II, and not Ang I, increased inflammatory gene expression in MMP8_KO SMCs. (**E**) ROS measurement in SMCs were performed using the DCF ROS/RNS Assay Kit. (**F**) ROS inhibition abolished Ang I-induced inflammatory gene expression in SMCs. Serum-starved WT or MMP8_KO SMCs were incubated with 25 μg/mL BAPN with or without 10 nM Ang I, in the absence or presence of diphenyleneiodonium chloride (DPI, 10 µM), for 24 h. Total RNAs were extracted and subjected to RT-qPCR analysis. (**G**,**H**) ROS inhibition abolished BAPN/Ang I-induced SMC apoptosis. Serum-starved WT or MMP8_KO SMCs were subjected to the indicated treatments for 48 h, followed by CCK-8 assays (**G**) or TUNEL staining analysis (**H**), respectively. Data presented here are the mean ± SEM of five independent experiments (*n* = 5). * *p* < 0.05 (versus vehicle for Ang II in **A**–**C**, BAPN/vehicle in **D**,**E**, BAPN/vehicle/DMSO in **F**, or vehicle/DMSO in **G**,**H**); ^#^ *p* < 0.05 (versus vehicle for BAPN in **A**–**C**, WT in **D**,**E**, or BAPN/Ang I/DMSO in **F**–**H**); two-way ANOVA with a post-hoc Tukey test.

As previously described, decreased aortic inflammation was observed in MMP8_KO mice during BAPN-induced TAD development (Figure 3A–C). To study the potential involvement of MMP8 in BAPN/Ang II-induced SMC inflammation, we first examined if BAPN and/or Ang II could regulate MMP8 gene expression and activity in SMCs. Indeed, we found that incubation of SMCs with BAPN or Ang II alone could significantly upregulate MMP8 gene expression and activity. Additionally, the synergic effect of BAPN and Ang II on the MMP8 expression level and activity was observed in SMCs (Figure 6A,B). Moreover, similar regulatory effects of BAPN and Ang II on multiple inflammatory genes were observed in SMCs (Figure 6C). Importantly, we found that while Ang II significantly upregulated inflammatory gene expression in both WT and MMP8_KO SMCs, such gene upregulation was only observed in WT SMCs when cells were incubated with Ang I (Figure 6D). It is widely known that reactive oxygen species (ROS) generation is one of the main downstream effectors of Ang II signaling in vascular pathology [39], we wondered if a similar mechanism also underpinned MMP8-mediated aortic and SMC inflammation. Indeed, IF staining with both dihydroethidium (DHE) dye and 8-hydroxydeoxyguanosine (8-OHdG) antibody revealed a significantly lower ROS level in MMP8_KO aortas than that in WT aortas upon BAPN treatment (Figure S4). Similarly, we observed that while ROS generation was increased by Ang II in both WT and MMP8_KO SMCs, Ang I promoted ROS generation in WT, but not in MMP8_KO SMCs (Figure 6E). As expected, Ang I upregulated inflammatory gene expression in SMCs, which was abolished by the ROS inhibitor, diphenyleneiodonium chloride (DPI). Importantly, such a regulatory effect was lost in MMP8_KO SMCs (Figure 6F). As mentioned above, a lower number of SMC apoptosis was observed in MMP8_KO aortas (Figure 3D,E). To further explore the potential role of MMP8 in SMC apoptosis under TAD pathological conditions, serum-starved SMCs were incubated with BAPN, Ang I, and/or DPI alone or in combination. Data from cell viability (Figure 6G) and TUNEL staining (Figure 6H and Figure S4) showed BAPN and Ang I co-incubation significantly induced WT SMC apoptosis, which was rescued by ROS inhibition. However, no such effect was observed in MMP8_KO SMCs (Figure 6G,H and Figure S5). These data have provided clear evidence to support an important role of MMP8 in BAPN/Ang II-induced SMC inflammation and apoptosis.

### 3.8. MMP8 Is Increased in the Dissected Human Arteries and Serum from Patients with Acute TAD

**Figure 7:**
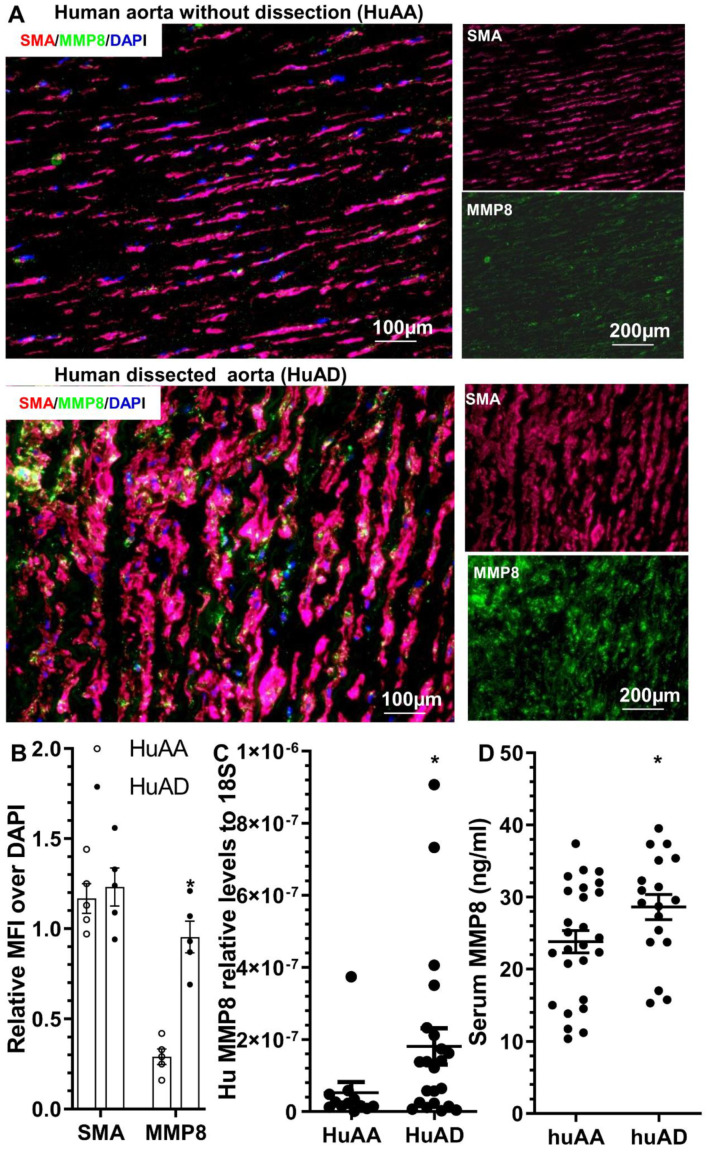
MMP8 detection in human ascending aorta with (HuAD) or without (HuAA) dissection. (**A**,**B**) Immunofluorescent staining showed increased expression levels of MMP8 protein in a dissected human ascending aorta. Representative images (**A**) and relative mean fluorescence intensity (MFI) (**B**) of MMP8 or SMA over DAPI staining are presented here. * *p* < 0.05 (*n* = 5, versus HuAA) (unpaired *t*-test). (**C**) RT-qPCR analysis showed increased MMP8 gene expression in a human ascending aorta with dissection. * *p* < 0.05 (*n* = 12 for HuAA or 22 for HuAD, versus HuAA) (Mann–Whitney *U* test). (**D**) ELISA analysis showed increased serum MMP8 levels in patients with acute TAD. * *p* < 0.05 (versus HuAA, *n* = 26 for HuAA and 18 for HuAD) (Mann–Whitney *U* test).

To study the potential involvement of MMP8 in human TAD, MMP8 expression was determined in the human ascending aortic tissues with or without dissection. Human ascending aortic tissue specimens and sera were obtained from TAD patients and normal healthy subjects, as previously described [27]. HE and EVG staining of the human aortas confirmed pathological changes in TAD in the dissected human arteries, such as disordered elastic lamellae with frequent elastin breaks, depletion of elastic fibers, and SMC loss (Figure S6). Immunostaining analysis showed an increased MMP8 protein expression in the dissected arteries compared to those without dissection (Figure 7A,B). Additionally, co-immunostaining revealed MMP8 was highly expressed in SMCs within the dissected human aorta (Figure 7A). Importantly, we observed increased aortic MMP8 gene expression (Figure 7C) and serum MMP8 level (Figure 7D) in patients with acute TAD, suggesting the potential involvement of MMP8 in the pathogenesis of human TAD.

## 4. Discussion

AD is a life-threatening aortic disease, with an incidence of 3–6 cases per 100,000 persons per year in Europe and the United States [1,2]. Currently, clinical management of patients with acute AD is mainly through surgical interventions, including hemiarch surgery, interposition aortic graft, aortic root replacement, and total arch replacement. Survival after surgical repair is still suboptimal, with a crude in-hospital mortality rate of 17.8% [40]. Unfortunately, no effective pharmacotherapeutic management is available, making it pertinent to identify novel therapeutics for AD treatment. In this study, we uncovered a novel function for MMP8 in the pathogenesis of TAD. MMP8 promotes TAD formation through converting Ang I to Ang II. Ang II generation at one hand promotes inflammatory cell migration/recruitment into the aortic wall, increasing aortic inflammation; on the other hand, Ang II also increases SMC apoptosis by modulating ROS generation, thereby promoting TAD formation/development.

MMP8 has been reported to play a causal role in multiple vascular diseases [14]. Particularly, we have provided comprehensive evidence to support a critical role for MMP8 in atherogenesis [11], angiogenesis [18], stem/progenitor cell recruitment/migration into atherosclerotic plaque [13], macrophage differentiation/polarization [8], SMC proliferation/migration and post-angioplasty restenosis [12], and adventitia stem/progenitor cell differentiation towards SMCs and their contributions to vascular injury-induced neointimal SMC hyperplasia [19]. Moreover, an increasing number of clinical epidemiological studies have also identified MMP8 as an independent risk factor for several cardiovascular diseases, such as coronary artery disease [41,42], unstable angina [43], ischemic stroke [44], unstable carotid plaques [45], acute myocardial infarction [41,46], and aortic aneurysm [47,48]. Regarding AD, clinical studies showed that significantly higher serum MMP-8 levels were detected in acute AD patients than in control subjects [49], that a prominent increase in plasma MMP8 level was reported in the acute phase of AD [50], and that combination of plasma MMP8 and D-dimer at individually suboptimal cutoffs could provide a better diagnostic value for acute AD under medical emergency [51]. Moreover, it has been reported that the C-799T polymorphism in the MMP8 promoter is significantly associated with susceptibility to disease progression in AD patients [52]. In line with these clinical findings, we also found increased levels of MMP8 in the dissected human arteries and serum from patients with acute TAD. Importantly, applying both genetic and pharmacologic strategies in this study, we have comprehensively confirmed a causal role for MMP8 in TAD formation, and provided clear evidence to prove the therapeutic potential of using MMP8 inhibitor in treating patients with acute TAD. Surprisingly, we found no significant difference in terms of survival rate between WT and MMP8_KO mice (Figure 1C), as well as WT mice treated with vehicle control and MMP8 inhibitor (Figure 2C), suggesting a redundant role for MMP8 in BAPN-induced aortic rupture or mortality. However, such an insignificant finding may be attributed to the low mortality rate induced by BAPN alone (30~40%). Accordingly, the potential effect of MMP8 on aortic rupture (sudden death) warrant further investigation using another acute TAD model, such as the BAPN/Ang II infusion model, in which a higher mortality (70%) was reported [4].

Interestingly, we observed that MMP8 was highly expressed in multiple aortic cells, including neutrophils, macrophages, and SMCs in BAPN-induced TAD (Figure 7 and Figure S2). MMP8 gene expression was also significantly activated by BAPN treatment in both ECs and SMCs (Figure 5G and Figure 6A). Importantly, our data showed that MMP8 could be produced by their respective cells and released into the extracellular space, as evidenced by the higher MMP8 enzymatic activity detected in cell culture supernatant (Figure 5H and Figure 6B), suggesting that in an in vivo disease setting of TAD, multiple cells (e.g., neutrophils, macrophages, SMCs and ECs) can interact with and regulate each other’s functions in an autocrine and/or paracrine manner. Indeed, we observed that an increasing number of neutrophils and macrophages accumulated within aortic adventitia before TAD onset (up to two weeks post-BAPN administration) (Figure 3A–C), and found that a large number of MMP8-expressing inflammatory cells were recruited/migrated to the media layer and/or dissection sites (three weeks post-BAPN administration) (Figure S2). Accordingly, we speculate that these recruited inflammatory cells as well as activated resident ECs/SMCs produce/secrete MMP8, which on the one hand directly cleaves/degrades collagens, the weakening aortic wall, but on the other hand may also act on SMCs and cause SMC dysfunction (apoptosis and disorganization, as shown by green dot line in Figure S2), thereby promoting TAD onset (red arrows in Figure S2). However, it is worth mentioning that one of the limitations in this study is that our current data do not allow us to comprehensively elaborate the potential contribution of MMP8 derived from different cells due to the lack of the conditional MMP8 gene knockout mice. Moreover, the nature of MMP8 as a secretory protein makes it extremely difficult to achieve such a purpose, since this type of protein can exert its cellular functions in both an autocrine and paracrine manner. Indeed, we have previously reported that adventitia stem/progenitor cells do not express MMP8, but macrophage-derived MMP8 promotes these stem/progenitor cells’ differentiation into SMCs, thus contributing to neointimal SMC hyperplasia [19].

Mechanistically, we have shown that MMP8 promotes TAD formation/progression through modulating the Ang I to Ang II conversion, and its downstream effectors such as VCAM1 gene expression in ECs and ROS generation in SMCs, respectively. We have previously demonstrated that MMP8 can directly cleave Ang I, mainly generating Ang II, and the resultant Ang I product upregulates VCAM1 gene expression in ECs, which enhances leukocyte rolling and adhesions on the endothelium, thereby increasing atherosclerotic plaque inflammation and growth [11]. Here, we found a similar mechanism underlying MMP8-mediated inflammatory cell migration/recruitment under TAD pathology. Specifically, we have provided several lines of evidence to support such a notion. We first found high inflammatory cell accumulation within WT, but not in the MMP8_KO aortic wall, preceding TAD onset. Second, we observed increased expression levels of Ang I but decreased expression levels of Ang II in MMP8_KO aortic tissues as well as plasma upon BAPN administration, confirming less Ang II generation in the absence of MMP8 under TAD pathology. Third, a much lower level of VCAM1 gene/protein expression was observed in MMP8_KO aortas. Fourth, MMP8_KO inflammatory cells exhibited a decreased cell invasion and transendothelial migration capacity compared to WT cells. Fifth, we showed that while Ang II could increase both WT and MMP8_KO monocytes transendothelial migration, the WT but not MMP8_KO monocytes’ transendothelial migration was significantly increased in response to Ang I incubation, confirming the importance of the Ang II being converted from Ang I by MMP8 during MMP8-mediated inflammatory cell migration. Sixth, we also demonstrated that Ang I upregulated VCAM1 gene expression in control ECs, but not in cells with MMP8 inhibition. However, Ang II could activate VCAM1 gene expression in ECs with or without MMP8 inhibition. Lastly, our data showed that VCAM1 gene activation in ECs is at least partially responsible for the Ang II-mediated monocytes’ transendothelial migration. Taken together, these data have collectively demonstrated that the Ang I/Ang II/VCAM1 signaling axis is one of the underlying mechanisms of MMP8-mediated inflammatory cell migration/recruitment into aortic walls during TAD formation. In line with previous observations [4,53,54], we demonstrate that accumulated inflammatory cells within aortic walls increases aortic inflammation and extracellular matrix remodeling, thereby promoting TAD formation. 

Importantly, apart from the abovementioned actions, Ang II has been reported as a critical regulator and trigger for AD formation and onset. Specifically, focal dissections in the outer convex aspect of the ascending aorta, intimal tear, and interlaminar hematoma were observed in over half of the Ang II-infused animals [55]. Interestingly, although Ang II infusion has been widely used for aortic aneurysm animal models, it has been proposed that Ang II-infused mice are more clinically relevant for the study of AD than for the study of abdominal aortic aneurysms [56]. It is well-accepted that the hypertensive effect of Ang II is one of the main underlying causes of Ang II-induced AD. A recent, large clinical epidemiological study, with over one million participants, showed that hypertension and elevated SBP and DBP are associated with a high risk of AD, and the AD risk is positively dose-dependent, even within the normal blood pressure range [57]. Such an important finding is further supported in the meta-analysis with over 4.5 million participants [57]. Consistent with this finding, we also observed a significant decrease in both SBP and DBP in MMP8_KO mice. Therefore, a lower TAD incidence observed in MMP8_KO mice could be attributed to a decrease in both SBP and DBP caused by a lower level of Ang II. 

Additionally, our data also suggest that protecting SMCs from Ang II/BAPN-induced apoptosis is another important mechanism that underpinned the lower TAD incidence observed in MMP8_KO mice. Through multiple in vivo and in vitro studies, we observed a much lower level of SMC apoptosis in MMP8_KO mice compared with WT mice under BAPN treatment. Further in vivo and in vitro observational and mechanistic studies revealed that Ang II-induced ROS generation was the main attributing factor to Ang II/BAPN-induced SMC apoptosis. Our findings are well aligned with the previous observations that ROS is the key mediator of Ang II signaling [58,59] and Ang II-induced vascular cell apoptosis [60] or aortic pathologies [61]. It has also been reported that Ang II induces SMC apoptosis by partially modulating Akt phosphorylation and membrane/soluble Fas ligand expression [62], or through an Ang II type 2 receptor- and GATA-6-dependent mechanism [63]. Therefore, it would be interesting to study if these molecular mechanisms also underpin SMC apoptosis induced by Ang II/BAPN in the context of TAD formation, which will be the main focus of our future investigations. Another important finding in this study is that decreased ROS generation is observed in MMP8_KO mice, which could be another contributing factor to the lower TAD incidence in MMP8_KO mice. This observation is consistent with the key finding reported in a previous study that endothelial cell-derived ROS play a critical role in the determination of the susceptibility of the aortic wall to Ang II-mediated aortic dissection [64]. 

## 5. Conclusions

Taken together, in the current study we report the important role of MMP8 in TAD formation and demonstrate that MMP8 promotes TAD formation by increasing inflammatory cell recruitment/migration into vascular walls, enhancing aortic inflammation and augmenting SMC apoptosis. MMP8 exerts these cellular functions at least partially through converting Ang I to Ang II, which upregulates VCAM1 expression, facilitating inflammatory cell adhesion and transendothelium migration, thereby increasing aortic inflammation. On the other hand, the generated Ang II promotes SMC apoptosis under a TAD pathological setting by increasing ROS generation. Importantly, Ang II also has a direct role in controlling blood pressure. Therefore, increased aortic inflammation, more SMC death, and higher blood pressure act in concert with other MMP8 cellular actions, including collagen degradation, eventually promoting TAD formation and progression. Thus, data from this study provide new insight into the biological molecules and relevant mechanisms involved in the pathogenesis of TAD, and highlights the therapeutic potential of MMP8 inhibition in patients with TAD.

## Data Availability

The data that support the findings of this study could be found in our manuscript and supplementary data or are available from the corresponding author upon reasonable request.

## Supplementary Materials

The following supporting information can be downloaded at: https://www.mdpi.com/article/10.3390/cells11203218/s1, Table S1: Baseline Demographic Data for the collection of human arterial specimens from patients with (HuAD) or without (HuAA) acute TAD. Table S2: Baseline Demographic Data for the collection of human serum from patients with (HuAD) or without (HuAA) acute TAD. Table S3: Primer sets used in the present study. Figure S1 MMP8 expression level was increased during BAPN-induced TAD formation. Figure S2 MMP8 expressing neutrophils were observed in the dissected sites. Figure S3 IgG control for Ang I and Ang II staining. Figure S4 Decreased reactive oxygen species (ROS) generation in aorta was observed in MMP8_KO mice. Figure S5 Representative images of TUNEL staining in SMCs with the indicated treatments. Figure S6 Representative images of H&E and EVG staining of human ascending aorta with (HuAD) or without (HuAA) dissection.
