# Supplementary material for: Targeted Inhibition of Matrix Metalloproteinase-8 Prevents Aortic Dissection in a Murine Model
Source: Cells. 2022 Oct 14;11(20):3218. doi: 10.3390/cells11203218 (PMC9600539; doi:10.3390/cells11203218)

SUPPLEMENTARY DATA

Targeted Inhibition of Matrix Metalloproteinase-8 Prevents Aortic Dissection

(Zhang et al. MMP8 in aortic dissection)

Supplementary table S1: Baseline Demographic Data for the collection of human arterial specimens from patients with (HuAD) or without (HuAA) acute TAD

Characteristic	HuAA (n=12)	HuAD (n=22)	P Value
Male sex	10 (83.3%)	19 (86.4%)	0.812
Age (year)	43.05±10.22	45.88±7.62	0.258
Obesity*	8 (66.7%)	15 (68.2%)	0.928
Diabetes	1 (8.3%)	2 (9.1%)	0.941
Hypertension	3 (25%)	18 (81.2%)	0.001
Drinking	3 (25%)	5 (22.7%)	0.881
Smoking	8 (66.7%)	15 (68.2%)	0.928
Family history of aortic diseases	0 (0%)	2 (9.1%)	0.529
Dyslipidemia	1 (8.3%)	3 (13.6%)	0.646

Note: *Obesity refers to BMI >25 kg/m^2. Fisher's exact test was used for statistical analysis, except age which was tested using Student's *t*-test.

Supplementary table S2: Baseline Demographic Data for the collection of human serum from patients with (HuAD) or without (HuAA) acute TAD

Characteristic	HuAA (n=26)	HuAD (n=18)	P Value
Male sex	20 (76.9%)	15 (83.3%)	0.716
Age (year)	44.89±8.69	56.72±7.99	0.339
Obesity*	14 (53.8%)	13 (72.2%)	0.346
Diabetes	2 (7.7%)	2 (11.1%)	1.0
Hypertension	2 (7.7%)	16 (88.9%)	<0.001
Drinking	5 (19.2%)	4 (22.2%)	1.0
Smoking	13 (50%)	14 (77.8%)	0.1143
Family history of aortic diseases	0 (0%)	2 (11.1%)	0.162
Dyslipidemia	2 (7.7%)	3 (16.7%)	0.386

Note: *Obesity refers to BMI >25 kg/m^2. Fisher's exact test was used for statistical analysis, except age which was tested using Student's *t*-test.

Supplementary table S3: Primer sets used in the present study (Mus, Mouse; Hu, human)

Gene names	Forward (5'-3')	Reverse (5'-3')	Application
18s (mus/hu)	AAACGGCTACCACATCCAAG	CCTCCAATGGATCCTCGTTA	Real-time RT-PCR
Mus MMP8	GTCCCAAGTGGACACACACT	TCACTTCAGCCCTTGACAGC	Real-time RT-PCR
Hu MMP8	AAAGAAAGCCAGGAGGGGTA	ACAGGAAAGGCCTTGGAAAT	Real-time RT-PCR
Mus MMP1	GCCAGAACCTGAGCTCAATTTAATA	GCCCATACTTTGCTGCCTTT	Real-time RT-PCR
Mus MMP13	GGAGCCCTGATGTTTCCCAT	GTCTTCATCGCCTGGACCATA	Real-time RT-PCR
Mus MCP1	CCCCAAGAAGGAATGGGTCC	TGCTTGAGGTGGTTGTGGAA	Real-time RT-PCR
Mus IL6	GTGGCTAAGGACCAAGACCA	TAACGCACTAGGTTTGCCGA	Real-time RT-PCR
Mus IL12β	AGTGACATGTGGAATGGCGT	CAGTTCAATGGGCAGGGTCT	Real-time RT-PCR
Mus VCAM1	TTCTGACGTGTGCTGCTATTGG	TTTGGCCCCCTCATTCCTT	Real-time RT-PCR
Mus Nos2	GCCACCAACAATGGCAACAT	TCGATGCACAACTGGGTGAA	Real-time RT-PCR

Supplementary Figures:

Figure S1. MMP8 expression level was increased during BAPN-induced TAD formation.

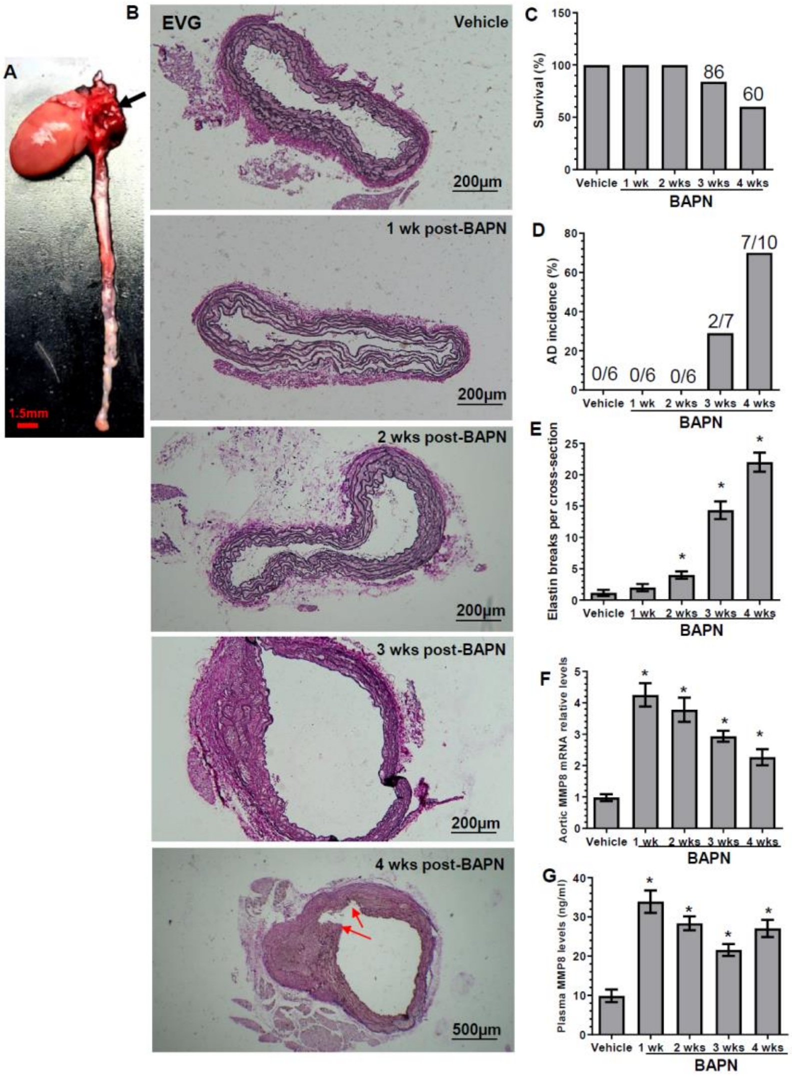

Three week-old mice were fed a normal diet and randomly administered with vehicle (water) or freshly prepared 3-aminopropionitrile fumarate (BAPN) solution dissolved in the drinking water (0.25%, wt/vol) for indicated times, thoracic aorta and plasma were collected and subjected to histological (A-E), RT-qPCR (F) and ELISA (G) analysis, respectively. Representative images showing the macroscopic features of isolated mouse aorta (A, arrow indicates location of TAD), Elastin Van Gieson (EVG) staining (B), and quantitative data of survival rate (C), TAD incidence (D), and Elastin breaks (E) were presented here. Note: TAD incidence was defined by the mice died from thoracic aortic rupture, and mice identified with one or more aortic pathologies (aortic intima tear (red arrows), false lumen, intramural haematoma). (F) RT-qPCR analysis of the thoracic aortic MMP8 expression. (G) ELISA detected plasma MMP8 levels. Data presented here are representative (A) or Mean±S.E.M (B-F) of six mice (n=6 mice). *P<0.05 (versus vehicle, one-way ANOVA with a post hoc test of Tukey's analysis).

Figure S2. MMP8 expressing neutrophils were observed in the dissected sites.

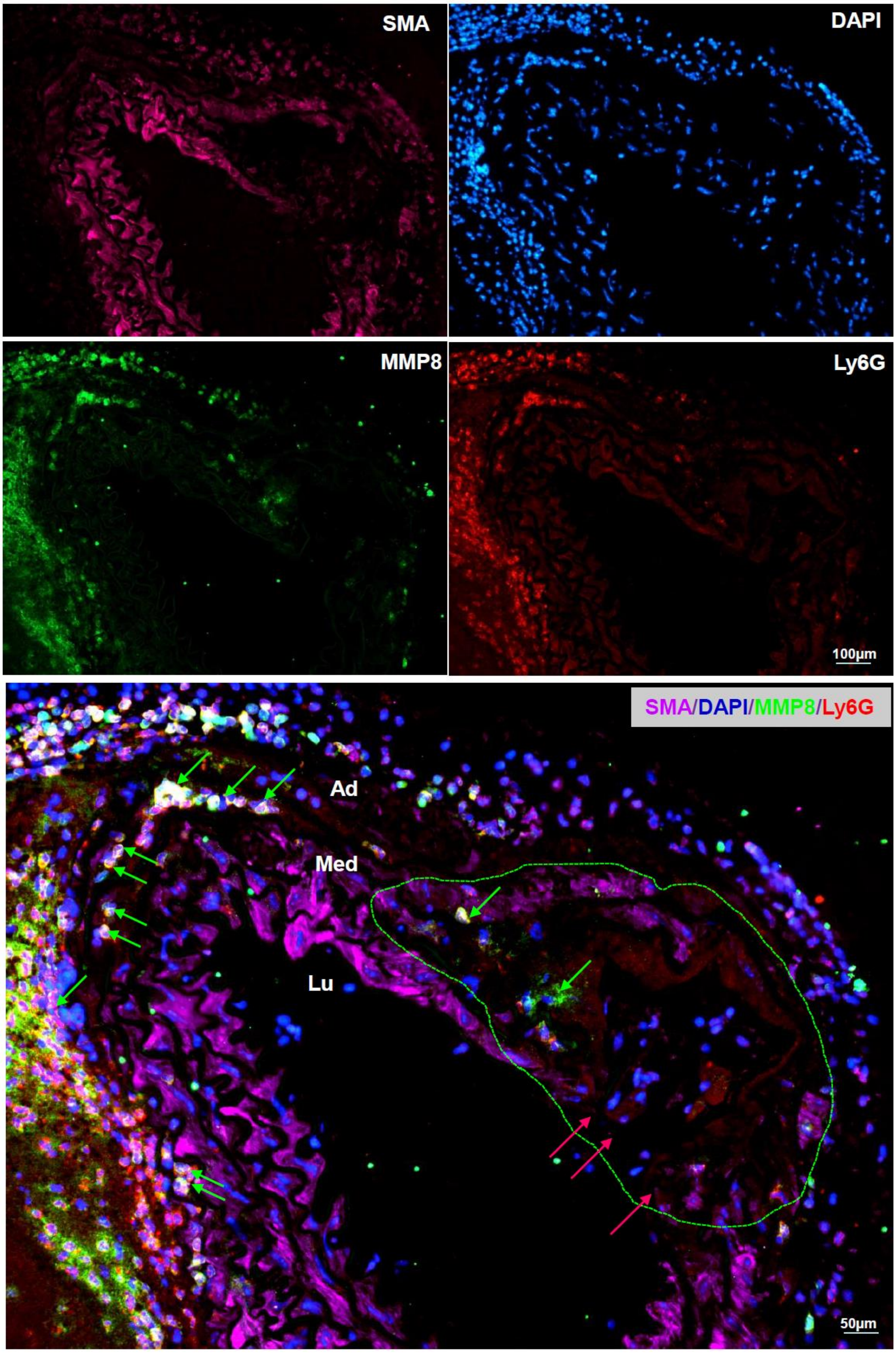

Three week-old WT mice were administered with BAPN in drinking water for three weeks, thoracic aortic tissues were collected and subjected to immunofluorescent staining analysis. Data presented here are representative images from six mice (n=6 mice). Green dot line indicates dissected regions, green arrows indicate MMP8 expressing neutrophils, and red arrows indicate AD, respectively. Lu, lumen; Med, media, Ad, adventitia.

Figure S3. IgG control for Ang I and Ang II staining.

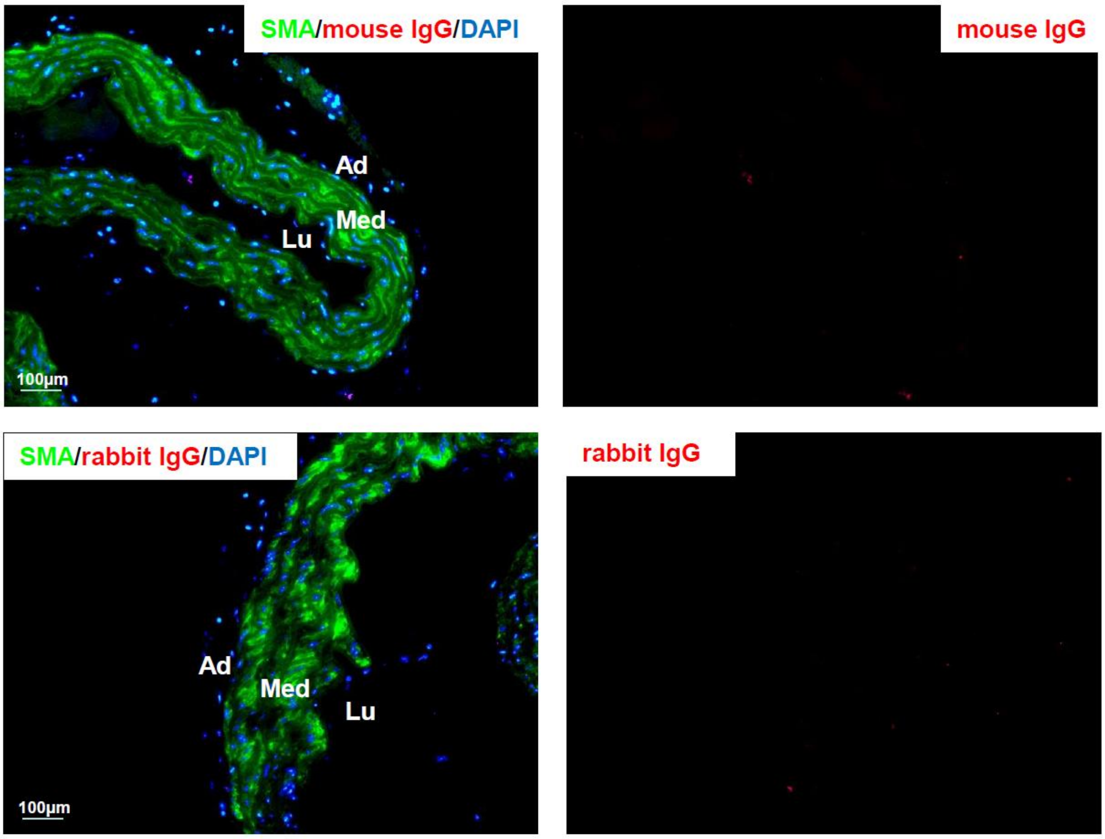

Three week-old mice were administered with BAPN in drinking water for two weeks, thoracic aortic tissues were collected and subjected to immunofluorescent staining analysis with anti-SMA antibody and indicated IgG control. Data presented here are representative images from six mice (n=6 mice). Lu, lumen; Med, media, Ad, adventitia.

Figure S4. Decreased reactive oxygen species (ROS) generation in aorta was observed in MMP8_KO mice.

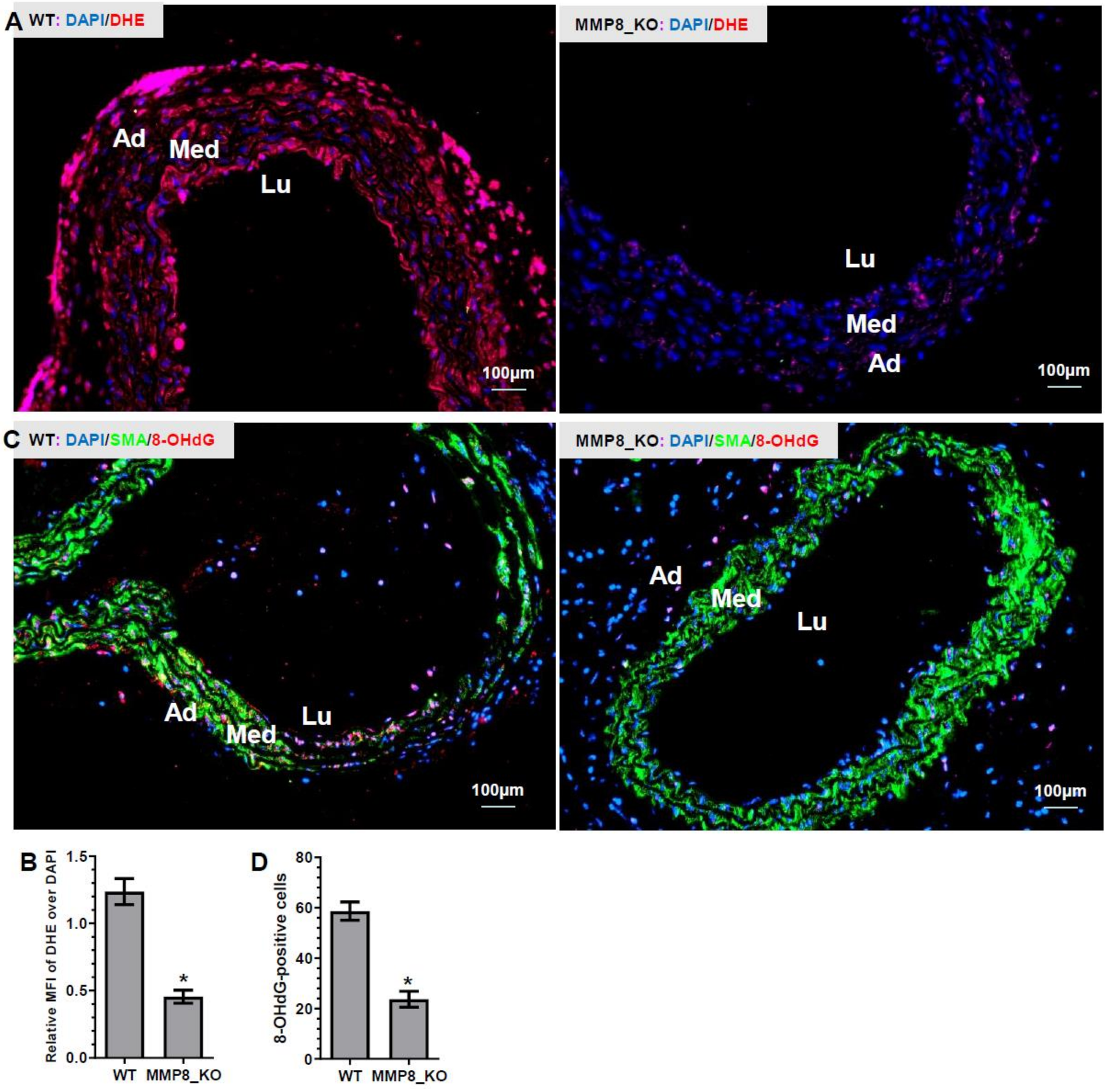

Three week-old WT and MMP8_KO mice were administered with BAPN in drinking water for two weeks, thoracic aortic tissues were collected and subjected to immunostaining analysis as indicated. (A-B) ROS detection by dihydroethidium (DHE) staining. (C-D) Immunofluorescent staining analysis of aortic tissues with antibodies against SMA and 8-hydroxydeoxyguanosine (8-OHdG). Data presented here are representative (A & C) or Mean±S.E.M of six (B & D) mice, respectively (n=6 mice). *P<0.05 (versus WT, unpaired *t*-test). Lu, lumen; Med, media, Ad, adventitia.

Figure S5. Representative images of TUNEL staining in SMCs with the indicated treatments. Green arrows indicate TUNEL-positive cells.

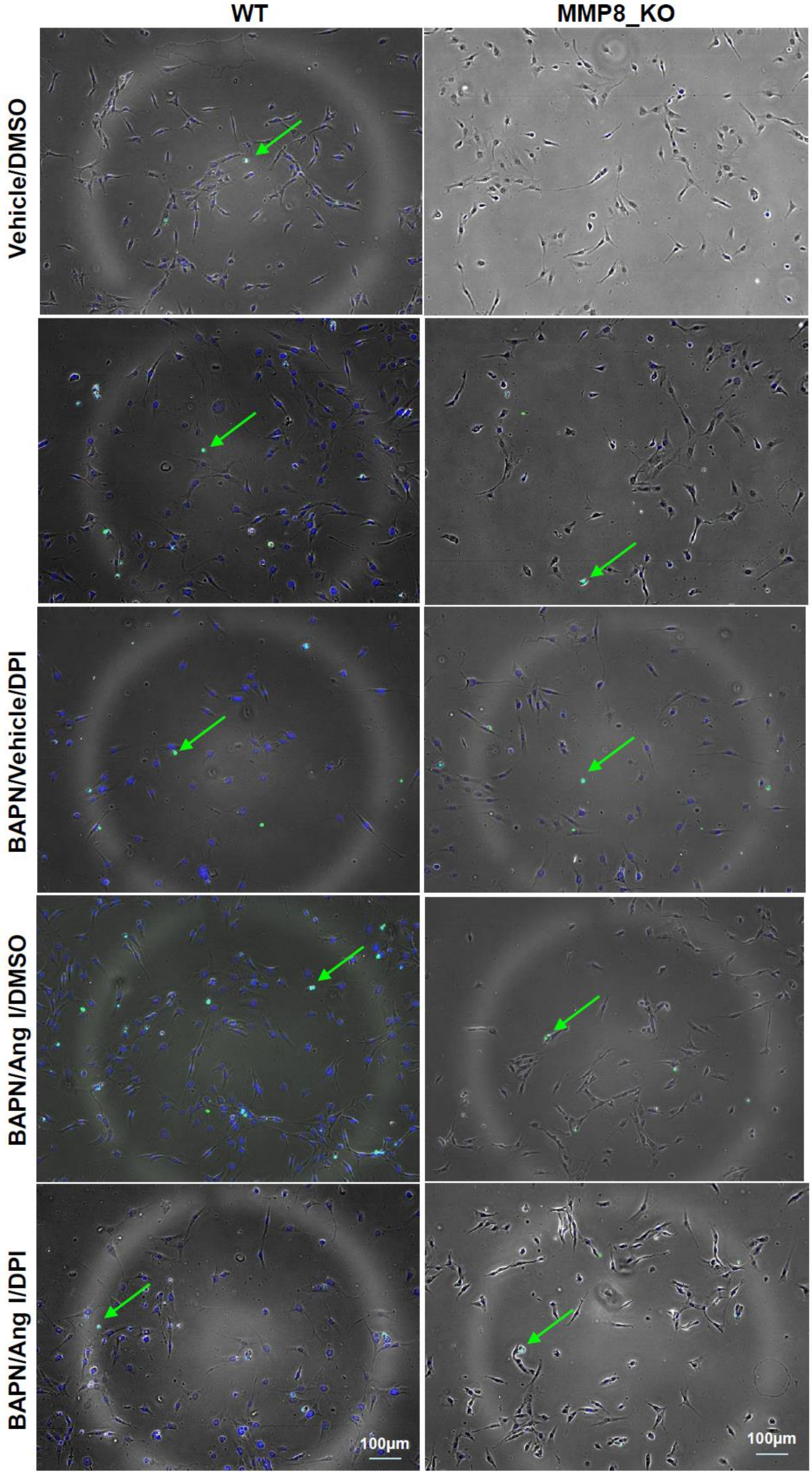

Figure S6. Representative images of H&E and EVG staining of human ascending aorta with (HuAD) or without (HuAA) dissection.

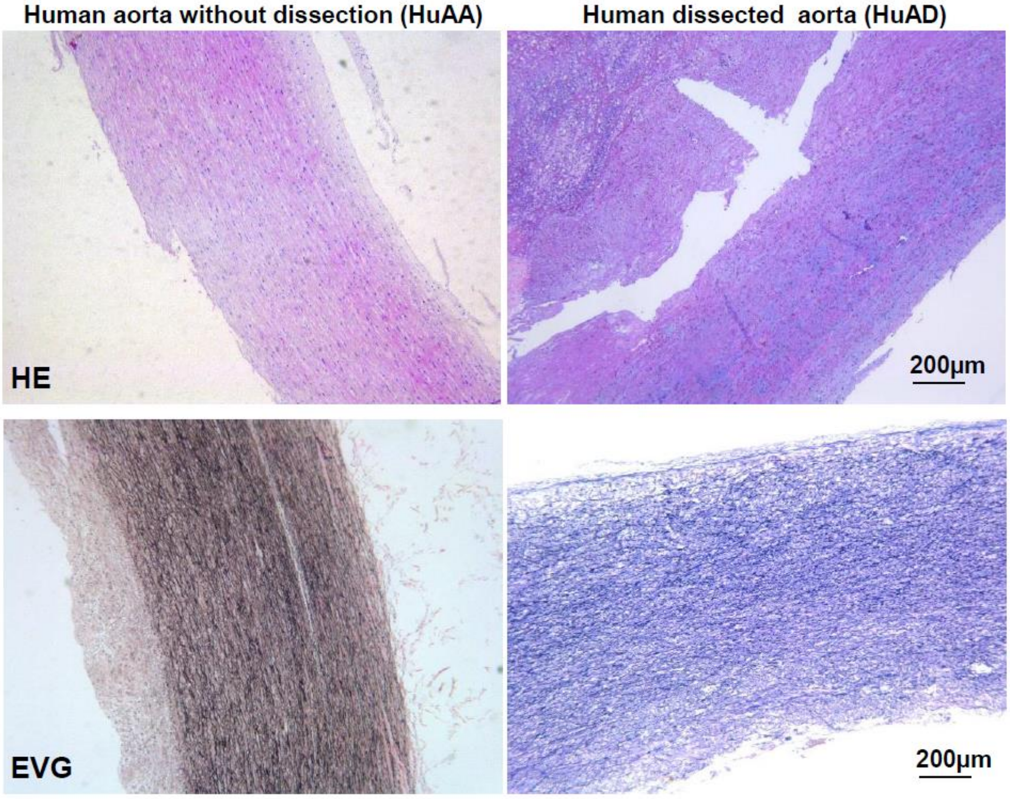

Supplement: Supplementary file 1 [file cells-11-03218-s001.zip › cells-1894033-supplementary.pdf]
